# Biophysical characterization of lutein or beta carotene-loaded cationic liposomes

**DOI:** 10.1039/d0ra05683a

**Published:** 2020-09-01

**Authors:** Nourhan S. Elkholy, Medhat W. Shafaa, Haitham S. Mohammed

**Affiliations:** Medical Biophysics Division, Physics Department, Faculty of Science, Helwan University Cairo Egypt phy.nsayed@gmail.com +201061782583; Biophysics Department, Faculty of Science, Cairo University Giza Egypt

## Abstract

The interactions between carotenoids and membrane constituents are vital for understanding the mechanism of their dynamic action. Lutein and beta-carotene were loaded separately into the bilayer of dipalmitoylphosphatidylcholine (DPPC) mixed at a molar ratio with l-α-phosphatidylethanolamine derived from sheep brain (cephalin) and stearylamine (SA) to form cationic liposomes. The molecular interaction between lutein or beta-carotene with cationic liposomes was studied using transmission electron microscopy (TEM), dynamic light scattering (DLS), differential scanning calorimetry (DSC), and Fourier transform infrared (FTIR) spectroscopy. Encapsulation efficiency (EE %) and *in vitro* drug release were determined. The DLS measurements confirmed the mono-dispersity of all samples. TEM results revealed that liposomal samples were oval-shaped and there was a change in their morphology and size upon encapsulation of lutein or beta-carotene. Beta-carotene was observed to adhere to the boundary surface within the liposomal assembly with external morphological alterations. EE% of lutein and beta-carotene exceeded 98.8 ± 0.3% and 87 ± 4%, respectively. Lutein doped with cationic liposomes shows better *in vitro* release stability (about 30%) than beta-carotene (about 45%) between the 3^rd^ and the 6^th^ hour manifested by lower leakage rate percentage of lutein which would lead to higher lutein retention. The incorporated lutein resulted in broadening and shifting of the major endothermic peak of the co-liposomes, while the incorporation of beta-carotene did not induce a noticeable shift. An FTIR study was employed to reveal structure alterations in the vesicles after the encapsulation of lutein or beta-carotene into liposomes. Encapsulation of lutein or beta-carotene into liposomes induced a change in the frequency of the symmetric and asymmetric CH_2_ stretching bands in the acyl chain that may influence the order of the membrane.

## Abstract 2

The interactions between carotenoids and membrane constituents are vital for understanding the mechanism of their dynamic action.

## Graphical abstract

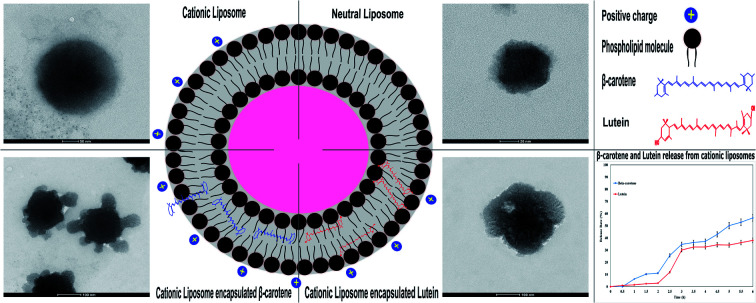

## 1. Introduction

Membranes are biological barriers which protect cells from the exterior environment. On the other hand, they selectively allow the permeation of ions and other small molecules to facilitate cellular signaling and promote cell homeostasis.^1–3^ Furthermore, they have the ability to selectivity degrade toxins (such as free radicals) that pass into the cell, which can result in damage to DNA and other cellular components. Carotenoids (plant natural pigments) have been found to support membrane defense through direct quenching and modulation of its physical properties.^3^

Carotenoids are lipophilic pigments which are found in the tissues of bacteria, plants and animals.^4,5^ Dietary carotenoids play an important role in vision, as antioxidants and as preventive actors in cancer, cardiovascular or skin diseases. They are biosynthesized in plants and microorganisms and play a role in photosynthesis and photoprotection. Xanthophylls are the class of carotenoids which consist of carotenoids contain hydroxyl groups such as lutein and zeaxanthin. Xanthophylls are more polar than hydrocarbons carotenoids such as beta-carotene and lycopene, which do not contain oxygen. The contribution of carotenoids at the cellular level is stringently related to their interaction with biomembranes. Studies were done to determine the location and the distribution of carotenoids in biological membranes and their inspiration on membrane properties.^6,7^

Liposomes are artificial membranous vesicles formed by phospholipids bilayers. Liposomes have long been receiving much consideration owing to their biocompatibility and interesting ability to carry hydrophobic and hydrophilic compounds. Due to their similarity with the bilayer structure of natural membranes, liposomes are used as a membrane model. The similarity between the liposomal and membrane bilayer core makes liposomes a very useful tool to inspect the consequence of carotenoids–membrane interactions.^8,9^

Our strategy was based on the development of highly lipophilic, positive charged and small agents, allowing them to diffuse successfully through the brain's endothelial cells. Liposomes vesicles, modified with a surface positive charge, made of pure l-α-dipalmitoylphosphatidylcholine (DPPC) mixed at molar ratio with l-α-cephalin derived from sheep brain were designed for this work since these phospholipids can mimic many characteristics of biological membranes in the brain. l-α-Cephalin is the major structural phospholipid in the brain, comprising 20–25% of total lipid; primarily localized to gray matter.

Cationic liposomes are magnificently used as drug delivery of therapeutic drugs and genes.^10,11^ Numerous studies have shown that these cationic nanocarriers are more effective vehicles for drug delivery to the brain than conventional, neutral, or anionic liposomes, maybe due to the electrostatic interactions between the cationic liposomes and the negatively charged cell membranes, enhancing nanoparticle uptake by adsorptive-mediated endocytosis.^12–14^ The present study was designed to use the positively charged liposomes as carriers of carotenoids in clinical therapy trials for brain-targeted drug delivery. In further planned work, this approach has to be extended to optimize the ideal liposome doped with carotenoids for crossing the blood–brain barrier, which has central implications for the dealing with neurological diseases.

The incorporation of carotenoids can affect the membrane fluidity,^15–17^ permeability,^16,18^ micropolarity,^15,16^ lipid peroxidation^19,20^ and thermotropic phase behavior.^21,22^ Carotenoids have distinct effects on the membrane properties, because of their various chemical structures. Overall assumption is that carotenoids having oxygen-containing groups in the ionone rings (xanthophylls) can exert rigidifying effects on the membrane fluidity, whereas the effects of hydrocarbon carotenoids having only carbon and hydrogen atoms (carotenes) are insignificant or opposite.^17,23,24^

Carotenoids have numerous health beneficial and nutritional effects and can decrease the risks of many disorders. Due to their high anti-inflammatory and antioxidant properties, they have been becoming interesting therapeutics for brain disorders, which may tender promising effects. However, they are supersensitive to processing and storage conditions and may lose their nutritional value before entering the human body. In addition, carotenoids have a low bioavailability and poorly delivered into central nervous systems due to the blood brain barrier (BBB). Nanoencapsulation is a strategy that can be used to maintain the original value of carotenoids and to enhance their functionality including bioaccessibility, digestibility, and controlled release.^25,26^

To our knowledge, there are no previous studies have been carried out on the biophysical interaction of carotenoids with cationic lipids especially those made of pure l-α-dipalmitoylphosphatidylcholine (DPPC) mixed at the molar ratio with l-α-cephalin derived from sheep brain since these phospholipids can mimic many characteristics of biological membranes in the brain.

The main objectives of the present study are to investigate how the molecular dynamic actions of carotenoids modulate the physical structural properties of cationic lipid membranes and to estimate the subtle perturbation of the lipid bilayer structure using transmission electron microscopy (TEM), dynamic light scattering (DLS), differential scanning calorimetry (DSC), Fourier transform infrared (FTIR) spectroscopy, as well as encapsulation efficiency and *in vitro* drug release measurements.

## 2. Materials and methods

### 2.1. Chemicals

**Fig. 1:**
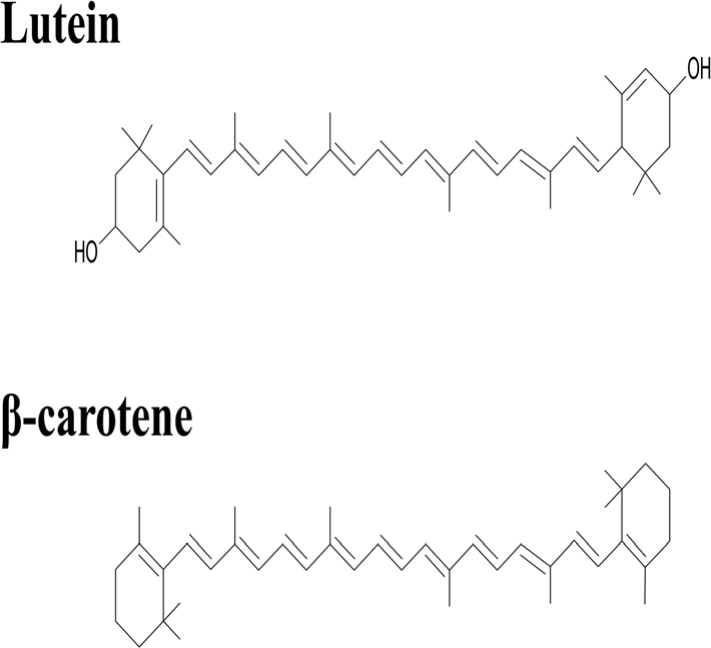
The chemical structure of lutein and beta-carotene.

**Fig. 2:**
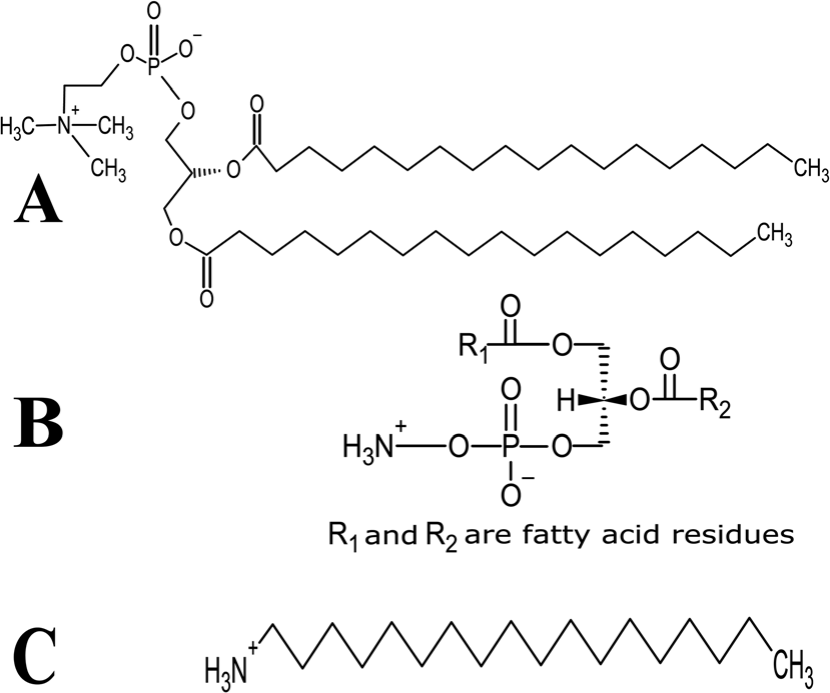
Schematic chemical structure of (A) DPPC, (B) l-α-phosphatidylethanolamine (l-α-cephalin) and (C) stearylamine (SA).

Lutein was purchased from S.C. Proplanta S.A. Cluj-Napoca Romania and beta-carotene was purchased from Sigma (St. Louis, Mo, USA). They were checked for purity by HPLC. The molecular weight of lutein is 568.42 and beta-carotene is 536.9. The molecular structures of the carotenoids used are shown in Fig. 1. Ethanol alcohol, absolute 99.9% was purchased from Daejung Chemicals (Seohaean-ro, Gyeonggi-do, Korea). l-α-Dipalmitoylphosphatidylcholine (DPPC) in powder form and of purity 99% with molecular weight of 734.039, l-α-cephalin (3-*sn*-phosphatidylethanolamine) from sheep brain with molecular weight of 691.515 of purity ≥ 98%, stearylamine (SA), molecular weight of 269.5 of purity 99% were purchased from Sigma (St. Louis, Mo, USA) are presented in Fig. 2, Tris base (hydroxymethyl) in powder form, molecular weight of 121.1, was purchased from CDH, New Delhi, India.

Solutions were prepared in distilled ultra-pure water. All other reagents and solvents used in this work were of HPLC grade.

### 2.2. Liposome preparation

Liposomes were prepared by the thin-film hydration method.^7,24,27^ Aliquots of DPPC mixed with l-α-cephalin were used at weight ratio (3 : 1 w/w). This ratio remained more thermally stable than any other lipid ratios as it was observed by DSC analysis (data not shown).

To prepare 10 mg of neutral empty liposomes (NL), DPPC and l-α-cephalin were mixed at weight ratio (3 : 1), accurately weighed amounts of 7.5 mg of DPPC and 2.5 mg of l-α-cephalin were added together and then dissolved in 10 ml ethanol in a round bottom flask. Ethanol is not particularly toxic and has a low order of acute toxicity by all routes of exposure.^28^

The liposome mixture was shacked well for a few minutes then vigorous vortexing took place to assure complete solvation. The organic solving was removed gradually using a rotary evaporator (Re-2010, Lanphan Zhengzhou, Henan, China) under vacuum produced by circulating water aspiration vacuum pump (SHB-III, USA Lab Equipment, USA) in a warm water bath at a temperature above the phase transition temperature of the suspended lipid (50°) at 60 rpm to produce a uniform thin film of lipid on the inner wall of the flask. The flask was then left under vacuum for 12 h to ensure the evaporation of all traces of ethanol. The lipid film was hydrated with 10 ml Tris buffer (pH 7.4 in 37 °C) in a water bath at 50 °C for 15 min at 60 rpm to form multilamellar vesicles (MLV). The MLV was sonicated by an ultrasonic homogenizer with titanium probe (model 150VT, BioLogics, USA) for 2 min at 50% amplitude with a pulse 90% to form the small unilamellar vesicles (SUV) of NL. Parallel to NL, cationic empty liposomes (CL) was prepared by addition of 0.5 mg of SA to the lipid composition to introduce a net positive charge at molar ratio = 1 : 7 for each type of lipid.

Carotenoid purity was checked before being used in experiments by TLC, HPLC and UV-Vis spectra in ethanol. A stock solution of lutein or beta-carotene was prepared where 10 mg of each carotenoid were dissolved in 10 ml of absolute ethanol. In order to characterize the carotenoid weight, UV-Vis absorbance of the carotenoids [100 μl] in an ethanol solution [2 ml] were measured by a spectrophotometer at 446 and 450 nm respectively.

By the calculation, the carotenoid weight is determined using the following formula to quantify the carotenoid extracts, known volumes of a carotenoid solution were used.
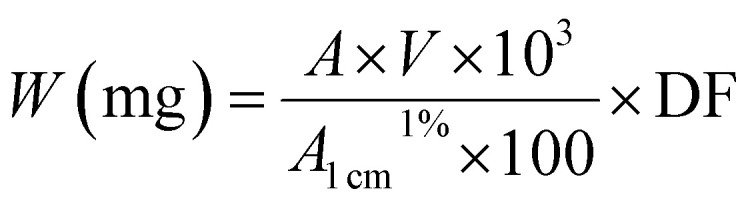
where *W* is the carotenoid weight, *A* is the optical density recorded [its value should be between 0.2–0.8]. *V* is the volume of the solution containing the carotenoid, is the specific extinction coefficient of each carotenoid in the solvent used [2550 for lutein and 2620 for beta carotene in ethanol] and DF is the dilution factor.^29^

Cationic lutein (CL-Lut) or beta-carotene (CL-Bc) liposomal samples were prepared at carotenoid to lipid molar ratio = 2 : 7 for each kind of lipid. In order to prepare 10 mg of CL-Lut and CL-Bc at carotenoid to lipid molar ratio of 2 : 7, corresponding amounts of 2.19 mg of lutein or 2.12 mg of beta-carotene were dissolved in 10 ml of absolute ethanol as a final volume, respectively. Then, ethanol was gradually removed using a rotary evaporator to form a dry film of lutein or beta-carotene at the round bottom of the flask. DPPC and l-α-cephalin mixed at a weight ratio of 3 : 1 (7.5 mg of DPPC and 2.5 mg of l-α-cephalin) with 0.5 mg of SA (to create a net positive surface charge) were dissolved in 10 ml ethanol and then added to the dry film of lutein or beta-carotene. Parallel to NL and CL, the mixture was evaporated and dried as before.

### 2.3. Encapsulation efficiency measurement

The determination of encapsulation efficiency (EE) was performed by extraction according to ref. 16. To obtain the free amount of carotenoids in suspension, aliquots of 1 ml carotenoid-loaded cationic liposomes and 3 ml ethyl acetate were mixed by vortexing vigorously for 3 min at ambient temperature. The mixed sample was centrifuged at 2000 rpm for 5 min for collecting the supernatant of the centrifuged sample. The above operation was repeated twice. Finally, the collected supernatant was combined in a tube and diluted with ethyl acetate. The free amount of carotenoid was respectively quantized spectrophotometrically at 450, and 446 nm for beta-carotene and lutein with ethyl acetate as the blank. Each experiment was carried out in a triplet. The total amount of carotenoid was expressed relative to the mass of the lipids through the carotenoid initial concentration (IC = mg carotenoid/mg lipids, % w/w).

The amount of carotenoid loading into the cationic liposomes was calculated as the difference between the total amount used to prepare loading liposomes and that recovered by extraction. The carotenoid encapsulation efficiency (EE, %) and loading content (LC, % w/w) were respectively calculated using the following equations:
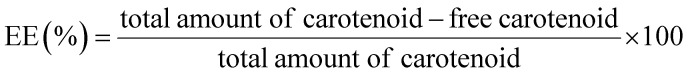

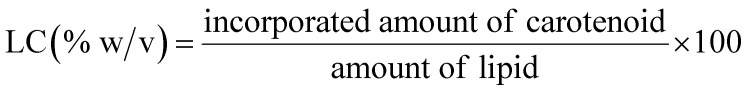


### 2.4. *In vitro* release studies

Phosphate buffer saline (PBS) was prepared with pH 7.4 of in 37 °C. A certain amount of cationic liposomal samples (5 ml) was mixed with 50 ml of PBS in a shaker and then stirred slowly in a water bath at 37 °C. At proper time intervals, an aliquot of the mixture was withdrawn and replaced with the same amount of PBS. The removed samples were extracted and analyzed spectrophotometrically in the same way for the determination of the free carotenoid as described above. The released carotenoids were calculated by subtracting the free amount of carotenoids initially prepared. The release rate was quantized as follows:
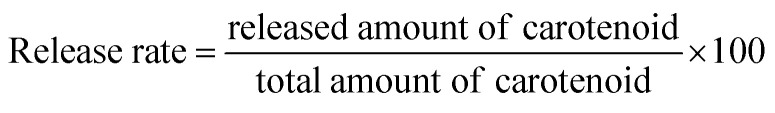


### 2.5. Liposome morphology by transmission electron microscopy

The size and morphology of the empty and encapsulated liposomes were studied using negative stain transmission electron microscope HR-TEM (Tecnai, G20, FEI, Netherland) operating at 200 kV. An aqueous solution of phosphotungstic acid stain (1% w/v) was used as a negative staining agent. Staining was followed by a 2 min wait at room temperature, removal of the excess solution with a filter paper, and then examination under the electron microscope. The liposome samples were first diluted (1 : 10) in Tris buffer pH 7.4 in 37 °C, and 20 μl aliquot was applied onto a transmission electron microscopy (TEM) grid (carbon coated copper grid). The solution was then left for 1 minute, and the excess was removed from the grid using filter paper.^30,31^

### 2.6. Dynamic light scattering measurements

The mean particle size and size distribution of freshly prepared empty, empty cationic and carotenoids-encapsulated cationic liposomes were determined by the dynamic light scattering using a particle sizing system (Nanotrac Wave II, Microtrac, USA) at 25 °C in Tris buffer (pH 7.4). The results were an average of three separate measurements.

### 2.7. Zeta potential measurements

The zeta potential of freshly prepared samples was determined in ultra-pure deionized water at 25 °C using Zetasizer Nano ZS, Malvern, UK. The results were an average of three individual measurements.

### 2.8. DSC measurements

Differential scanning calorimetry (DSC) experiments were carried out using (model DSC-50, Shimadzu, California, USA) calibrated with indium to investigate the thermal behavior of lyophilized samples of empty cationic and carotenoids-loaded cationic liposomes. Analyses are performed on 5 mg samples sealed in standard aluminum pans. The thermogram of each sample covers the 25–200 °C temperature range at a scanning rate of 3 °C min^−1^. Each sample was run in triplicates.

### 2.9. FTIR spectroscopy

FTIR spectra of lyophilized samples of CL, NL, CL-Lut and CL-Bc deposited in KBr disks are recorded on a Jasco FTIR-4100 spectrometer (Tokyo, Japan). Scanning is carried out at room temperature, in the range 4000–400 cm^−1^ at a speed of 2 mm s^−1^ and a resolution of 4 cm^−1^. Data were normalized and expressed as a mean of three independent samples.

## 3. Results

### 3.1. Encapsulation efficiency and loading content of lutein and beta-carotene in cationic liposomes

The EE% and LC% of carotenoid in cationic liposomal systems were analyzed separately by extraction and centrifugation. EE% values of lutein and beta-carotene, respectively, exceeded 98.8 ± 0.3% and 87 ± 4% even though the molar ratio to lipid was at 2 : 7. The LC of lutein and beta-carotene were exceeded 22% and 18% which are very close to their IC values (2 : 7 correspond to 28.57%), indicating that the added carotenoids can be effectively incorporated into a liposomal membrane Table 1.

**Table 1:** Encapsulation efficiency (EE%) and loading content (LC%) of lutein or beta carotene-loaded cationic liposomes as a function of initial concentration (IC%)

Parameter	CL-Lut	CL-Bc
IC%	28.57%	28.57%
EE%	98.8 ± 0.3%	87 ± 4%
LC%	22 ± 0.07%	18 ± 2%

### 3.2. Release rates of lutein and beta-carotene from liposomes formulation

**Fig. 3:**
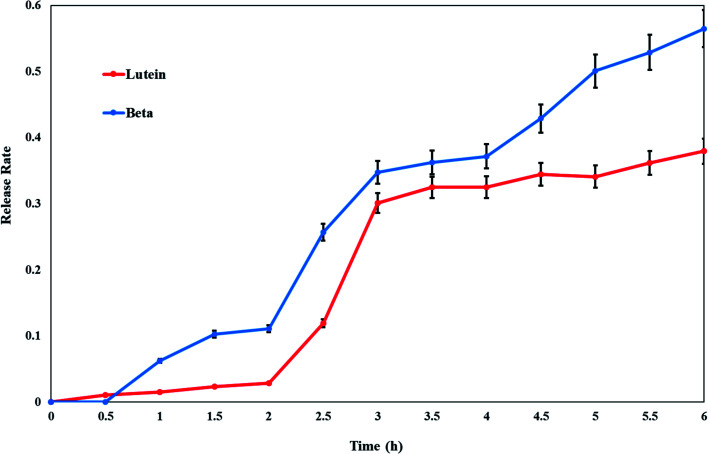
Release profile rates of lutein and beta-carotene from cationic liposomes. Data are expressed as mean ± S.E.

*In vitro* release of lutein and beta-carotene from the cationic liposomes was estimated to provide some indications of drug delivery performance. Lutein or beta-carotene leakage from liposomes formulation imparted with the positive surface charge was examined *versus* time in hours by the incubation in PBS at 37 °C. By further investigation of Fig. 3, it is clear that an initial phase of sustained drug release is apparent for either lutein or beta-carotene during the first 2 hours. At temperature 37 °C the formulation of CL-Lut or CL-Bc is fairly stable, releasing approximately 5–10% over the course of 2 hours followed by relatively rapid release rates (30–40%) over the next hours. The *in vitro* release results and the encapsulation efficiency revealed that lutein with the highest encapsulation efficiency (low leakage ability) showed the lowest drug release percentage.

The kinetic analysis of results revealed that lutein or beta-carotene was adopted first order kinetics when released from cationic liposomal forms as summarized in Table 2.

**Table 2:** Summarized data obtained for the release kinetic analysis for cationic liposomes encapsulated by lutein and beta carotene

Drug	Zero order		1^st^ order		Diffusion	
	Release score	Correction coefficient	Release score	Correction coefficient	Release score	Correction coefficient
Lutein	0.858	0.926	0.870	−0.933	0.865	0.930
Beta carotene	0.969	0.985	0.979	−0.990	0.967	0.983

### 3.3. TEM results

**Fig. 4:**
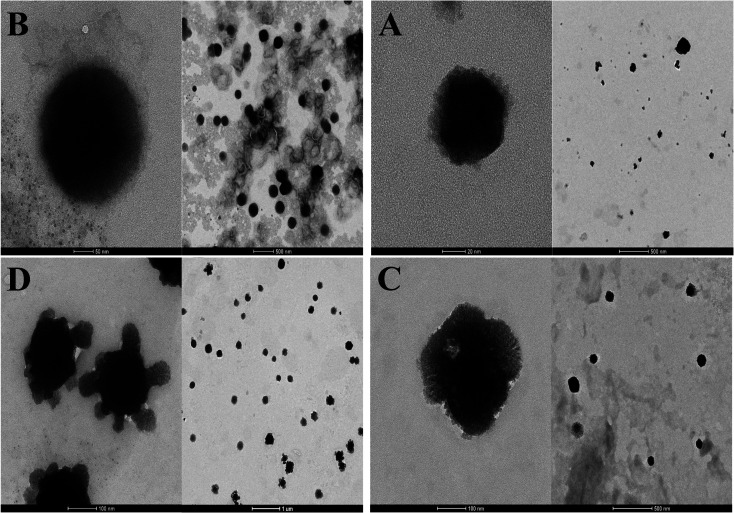
TEM images NL (A), CL (B), CL-Lut (C) and CL-Bc (D).

TEM images showed that the morphology of all liposomes prepared in this work was nearly oval-shaped, well dispersed and less aggregated for empty and encapsulated vesicles as shown from Fig. 4. The size of NL which is not imparted with the positive or negative surface charge was concentrated around 63.25 ± 49.75 nm while CL was in the range of 120 ± 114.8 nm (Fig. 4A and B). CL-Lut was in the range of 155.86 ± 125.6 nm (Fig. 4C). In the TEM image of CL-Bc was in the range of 225.54 ± 78.72 nm (Fig. 4D).

### 3.4. Size distribution of liposomes encapsulated carotenoids

**Fig. 5:**
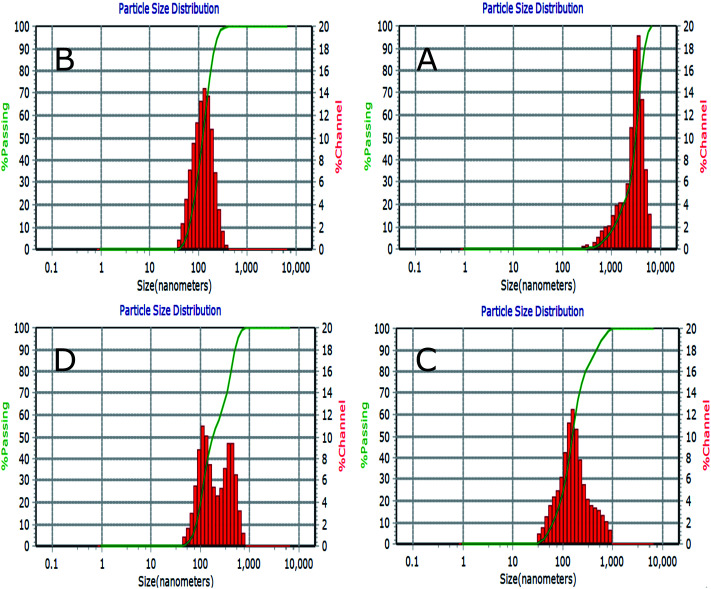
Liposomes size distribution measured by dynamic light scattering (DLS) for NL (A), CL (B), CL-Lut (C) and CL-Bc (D).

Fig. 5 represents a typical narrow size distribution graph for NL, CL, CL-Lut, and CL-Bc. Fig. 5A shows the size distribution of NL was concentrated around 3060 ± 1392 nm mean size diameter with 0.321 PDI. It is worth noting that the lack of surface charge may increase the aggregation of liposomes, thus may increase their size. In Fig. 5B a decrease in mean size diameter of CL to 123.5 ± 58.7 nm with 0.194 PDI.

The incorporation of lutein into CL resulted in an increase in the calculated mean size diameter from 123.5 ± 58.7 of CL nm to 157.9 ± 90.5 nm of CL-Lut with 0.361 PDI (Fig. 5C). Upon the encapsulation of beta-carotene into CL, the mean vesicle sizes were significantly increased to be in the range of 249.5 ± 50.7 nm with 0.396 PDI (Fig. 5D).

### 3.5. Zeta potential results

The results of zeta potential measurements of various liposomal formulations in the presence and absence of SA are summarized in Table 3.

**Table 3:** Summarized data obtained for transmission electron microscopy (TEM), the dynamic light scattering (DLS) by volume with polydispersity index (PDI) and the zeta potential for NL, CL, CL-Lut and CL-Bc

Sample	TEM	DLS		Zeta potential
	Mean size ± SD	Mean size ± SD	PDI	Mean zeta potential ± SD
NL	63.25 ± 49.75	3060 ± 1392	0.321	−22.38 ± 0.75
CL	120 ± 114.8	123.5 ± 58.7	0.194	36.33 ± 1.70
CL-Lut	155.31 ± 125.6	157.9 ± 90.5	0.361	38.03 ± 4.78
CL-Bc	225.54 ± 78.72	249.5 ± 50.7	0.396	34.40 ± 3.88

### 3.6. DSC results

**Fig. 6:**
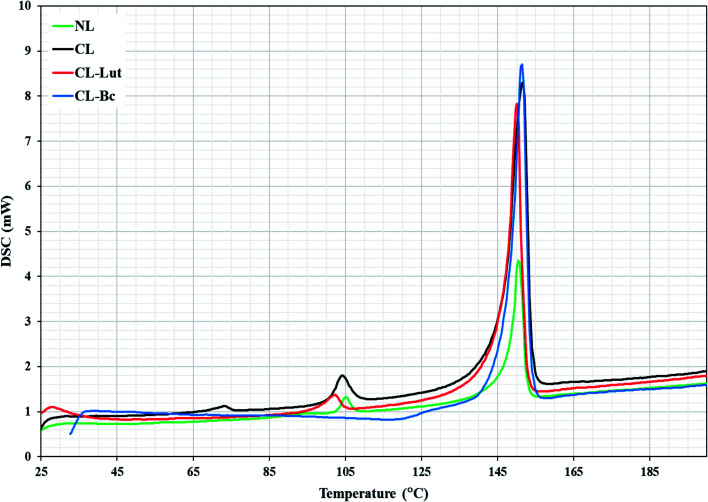
DSC diagrams of NL, CL, CL-Lut, CL-Bc.

DSC is a highly sensitive technique shows only subtle perturbation to the cooperative behavior of the membrane system in the absence or presence of foreign biomolecule as well as presents the thermotropic properties of many different biological macromolecules and extracts.^21,22^ DSC determines the thermal behavior of lipid bilayers and of lipidic drug delivery systems by measuring changes in the phase transition of the lipid bilayer due to altered interactions occurring between the encapsulated carotenoids and liposomes assemblies. NL showed two transition phases, the pre-transition phase (*T*_P_ = 105 °C), which indicates the transition from tilted gel phase to ripple gel phase and it is closely related to the phospholipid polar region (Fig. 6). The second is the main transition phase (*T*_m_ = 150.4 °C), that is known as the transition from ripple gel phase to the liquid crystalline phase and it is related to phospholipid acyl chains.^32^CL showed three transition phases. The first two phases were the pre-transition phases (*T*_P_ = 73, 104 °C) and the main transition phase (*T*_m_ = 151 °C).

The present DSC results showing that the encapsulation of lutein into the cationic liposomes resulted in a decrease in the (*T*_P_ = 102.8 °C) of the second peak related to CL to lower temperature. The first pre-transition temperature peak for CL-Lut disappeared. In addition, the present study showed that the encapsulation of lutein into cationic liposome resulted also in a decrease in the main transition temperature (*T*_m_ = 150.1 °C) of CL.

The incorporation of beta-carotene into cationic liposomes did not cause any noticeable shift to the major characteristic endothermic peak of CL that exists at 151.4 °C, however, its intensity was markedly increased. The pre-transition temperature is disappeared for the CL-Bc. Table 4 summarized data obtained for phase transition temperatures of NL, CL, CL-Lut and CL-Bc assemblies from DSC measurements.

**Table 4:** Phase transition temperatures of NL, CL, CL-Lut and CL-Bc assemblies obtained from DSC measurements

Transition peak	Temperature (°C)			
	NL	CL	CL-Lut	CL-Bc
1^st^ pre-transition	—	73.22	—	—
2^nd^ pre-transition	105	104	102.8	—
Main transition	150.4	151.4	150.1	151.4

### 3.7. FTIR results

**Fig. 7:**
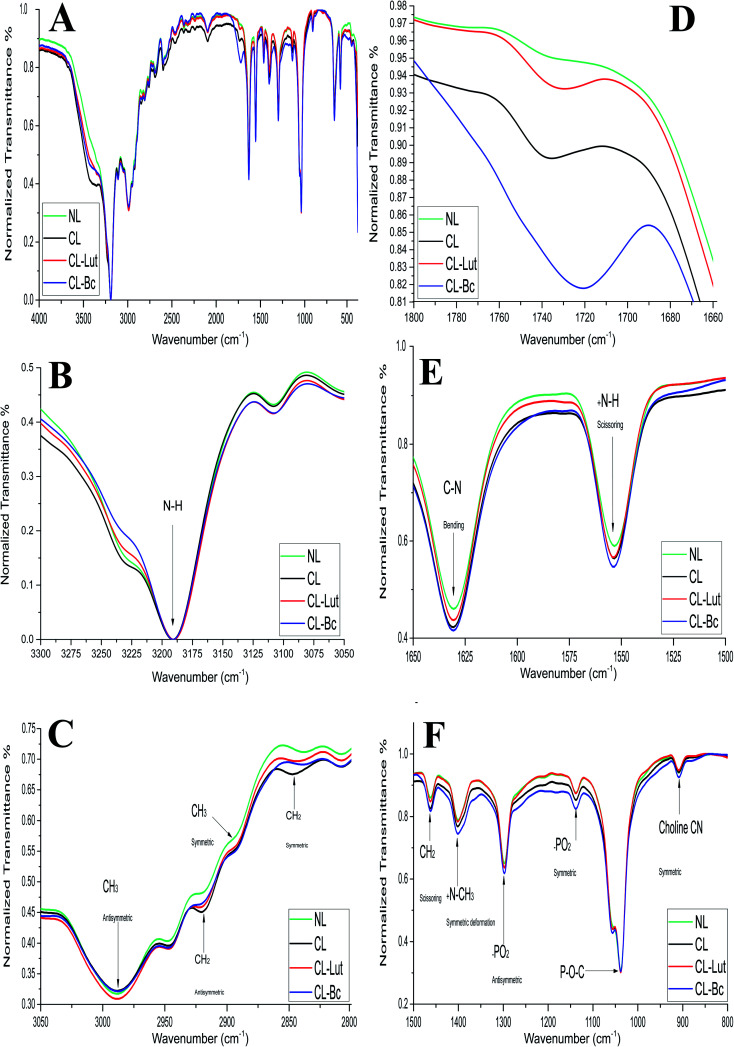
FTIR spectra of NL, CL, CL-Lut and CL-Bc samples: (A) all FTIR spectra in (4000 to 400 cm^−1^) region, (B) FTIR spectrum for the NH stretching vibration band in the region of (3300 to 3050 cm^−1^), (C) FTIR spectrum for the CH_2_ and CH_3_ symmetric and antisymmetric stretching vibration bands in the region of (3050 to 2800 cm^−1^), (D) FTIR spectrum for the carbonyl (C=O) stretching vibration band in the region of (1800 to 1650 cm^−1^), (E) FTIR spectrum for the C–N bending vibration and ^+^NH scissoring vibration bands in the region of (1650 to 1500 cm^−1^), and (F) FTIR spectra at the region (1500 to 800 cm^−1^) of NL, CL, CL-Lut and CL-Bc samples.

FTIR spectra of NL and CL compared with CL-Lut and CL-Bc samples in the region of 4000–400 cm^−1^ are depicted in (Fig. 7A). The spectrum displays the main characteristic bands, especially those due to the symmetric and antisymmetric PO_2_^−^ stretching vibrations at 1137.8 cm^−1^ and 1297.86 cm^−1^, respectively, the carbonyl stretching vibration C=O within the range of 1720–1740 cm^−1^ and the symmetric and antisymmetric stretching vibrations of the CH_2_ in the acyl chain within the region of 2800–2855 cm^−1^ and 2920–2924 cm^−1^, respectively.^33–49^

The detailed spectral analyses are performed in five distinct wavenumber regions, namely 3300–3050 cm^−1^ (Fig. 7B), 3050–2800 cm^−1^ (Fig. 7C), 1800–1660 cm^−1^ (Fig. 7D), 1660–1500 cm^−1^ (Fig. 7E), and 1500–800 cm^−1^ (Fig. 7F), since identifiable Raman bands are observed mainly in these regions only.

Addition of positive charge to form CL induces significant shift at the frequency of the symmetric CH_2_ stretching bands in the acyl chain towards higher wavenumber (from 2837.74 to 2847.3814 cm^−1^) when compared with NL (Table 5), while the asymmetric CH_2_ stretching bands in the acyl chain didn't change.

**Table 5:** The chemical shifts observed for lutein or beta carotene after the incorporation into cationic liposomes

Peak assignment	Wavenumber (cm^−1^)	Wavenumber (cm^−1^)			
		NL	CL	CL-Lut	CL-Bc
Symmetric stretching vibration of the CH_2_ in the acyl chain	(2800–2855)	2837.7391	2847.3814	2844.4887	2838.7033
Anti-symmetric stretching vibration of the CH_2_ in the acyl chain	(2920–2924)	2920.6632	2920.6632	2921.6274	2921.6274
CH_2_ scissoring vibration	(1457–1466)	1461.7779	1462.7422	1462.7422	1461.7779
Terminal CH_3_ symmetric stretching vibration	(2870–2900)	2893.6646	2891.7362	2891.7362	2891.7362
Terminal CH_3_ antisymmetric stretching vibration	(2957–2990)	2988.1595	2988.1595	2988.1595	2988.1595
Carbonyl stretching vibrations (C=O)	(1720–1740)	1735.6202	1735.6202	1729.8348	1721.1567
Symmetric ^−^PO_2_ stretching vibration	(1000–1260)	1137.7955	1137.7955	1137.7955	1137.7955
Anti-symmetric PO_2_ stretching vibration	(1200–1300)	1297.8583	1297.8583	1297.8583	1297.8583
Aliphatic phosphates stretch (P–O–C)	(900–1050)	1038.4794	1038.4794	1038.4794	1038.4794
CN symmetric stretching of choline	(904–930)	909.2722	909.2722	909.2722	908.3079
^+^N–CH_3_ symmetric deformation	(1396–1405)	1400.067	1401.0312	1401.0312	1401.0312
C–N bending vibration	(1630–1653)	1630.5188	1630.5188	1630.5188	1630.5188
^+^NH scissoring vibration	(1550–1559)	1553.3801	1553.3801	1553.3801	1553.3801
N–H stretching vibration	(3190)	3190.649	3190.649	3190.649	3190.649

Encapsulation of lutein or beta-carotene into the cationic liposomes induced the change in the frequency of the symmetric CH_2_ stretching bands. The peak at 2847.38 cm^−1^ for CL was shifted towards lower wavenumber 2844.49 and 2838.7 cm^−1^ for CL-Lut and CL-Bc, respectively. Interestingly the signal intensity became more intensive for CL-Lut or CL-Bc.

The interaction with the glycerol backbone near the head group of phospholipids in the interfacial region, the C=O stretching band is analyzed. The wavenumber variation of this band is shown in Fig. 7D. The wavenumber value of C=O group wasn't affected by SA extension nevertheless; it was shifted to lower values from 1735.62 cm^−1^ of CL to 1729.83 and 1721.16 cm^−1^ of CL-Lut and CL-Bc, respectively.

The interaction between lutein/beta-carotene and the head group of cationic liposomes with respect to NL and CL was monitored by means of the PO^2−^ symmetric and antisymmetric stretching vibrations band, which are located at 1137.80 and 1297.86 cm^−1^, respectively.^43,44^Fig. 7F shows the PO^2−^ symmetric and antisymmetric stretching band for NL, CL, CL-Lut and CL-Bc. As can be seen from Fig. 7F, the wavenumbers maintain unchanged in all samples. Symmetric stretching band of choline CN–(CH_3_)_3_ at 909.27 cm^−1^ is shifted to lower wavenumber 908.3 cm^−1^ for CL-Bc, whereas adding positive charge result in a shift of symmetric deformation band of choline to higher wavenumber from 1400.067 cm^−1^ to 1401.0312 cm^−1^.

## 4. Discussion

Liposomal formulations have been developed primarily to extend drugs circulating lifetime, increase the efficacy of drugs by increasing their deposition, and reduce drug toxicity by avoiding normal and healthy tissues. In the present work, cationic liposomes were prepared to encapsulate carotenoids rather than neutral liposomes. Liposomes with no net charge may increase the liposomal aggregation and consequently reducing their physical stability. Moreover, the less interaction of neutral liposomes with cells causes drug release from the liposomes in the extracellular space.^46,47^ The presence of a charge on the surface induces electrostatic repulsion among liposomes by creating a positive or negative zeta-potential preventing their aggregation and flocculation. A high electrostatic surface charge could promote the interaction of liposomes with cells.^50,51^ Therefore, several studies emphasized the potential use of charged liposomes for biomedical applications.^52–54^ In particular, studies focused on positively charged liposomes, due to the encouraging results obtained *in vitro* and *in vivo* experimentations.

The highest encapsulation efficiency percentage (EE%) is exerted by CL-Lut (98.8 ± 0.3%) followed by CL-Bc (87 ± 4%). So, lutein maintained stable to a higher extent than beta-carotene. The higher stability of lutein to bleaching is probably indicated by its distribution into two differently oriented pools within the membrane.^55^ In addition, lutein has one less conjugated double bond than does the other carotenoids, which may account for its slower reactivity to oxidation. Conclusively, lutein is the best incorporated and suited better into cationic liposomes. The results of the *in vitro* lutein or beta-carotene release at various time intervals, in the present study, has indicated that the maximum release (50%) occurred at last two hours and less than 10% release occurred at the first two hours. It can be observed that lutein doped with cationic liposomes shows better *in vitro* release stability (about 30%) than beta-carotene (about 45%) between the 3^rd^ and the 6^th^ hour manifested by lower leakage rate percentage of lutein which would lead to the higher lutein retention. Additionally, the percentage of beta-carotene released was approximately 60% over a 6 hours period.

Lutein vesicles show significant better *in vitro* release stability (*P* < 0.01) and lower leakage rate manifested in higher drug retention than that of CL-Bc. These results could be explained by the existence of two orthogonally oriented pools of lutein in lipid bilayer membrane^53,54^ resulting in higher stability than beta-carotene and consequently lower leakage rate. Lutein orientates vertically with respect to the membrane through hydrogen bonding between their polar end groups and the membrane's polar region.

Additionally, it is noted that the *in vitro* release results are consistent with those of the encapsulation efficiency, lutein with the highest encapsulation efficiency (low leakage ability) showed the lowest drug release percentage. These results could be explained by the fact that the presence of lutein in the bilayers below the phospholipid *T*_m_, which modulates membrane fluidity by restricting the movement of the relatively mobile hydrocarbon chains, reducing bilayer permeability, and decreases the efflux of the encapsulated drug, resulting in prolonged drug retention.

TEM results revealed that beta-carotene was detected at the boundary surface within the liposomal assembly which indicates that the liposomes may be physically associated with beta-carotene at the surface with disturbing the membrane packing property. In the TEM image of CL-Lut, the size of liposomes that entrap lutein increased significantly from 120 ± 114.8 nm to 155.86 ± 125.6 nm. It is supported that lutein and beta-carotene could be inserted in the hydrophobic region of the bilayer and these findings are in good agreement with the data observed by the DSC and FTIR results.

Dynamic light scattering (DLS) of a solid/liquid system, is a technique that is used in particle size measurement. Size is an important physical factor when dealt with the *in vivo* or *in vitro* environment. Nanomaterials and nano drug delivery system improve the bioavailability of the entrapped drug according to their size which match cells dimensions. Polydispersity index (PDI), is a number fit to correction data. PDI is a dimensionless and scaled such that values smaller than 0.05 are rarely seen other than with monodisperse standards. PDI effectively accounts for particle homogeneity of a colloidal suspension.

DLS results emphasize that the presence of charge may prevent aggregation of liposomes and consequently may decrease their size. The results of the particle size depicted by TEM are confirmed with results obtained by DLS measurements. As beta-carotene was observed at the boundary surface within liposomal assembly surface, the molecule of beta-carotene tends to be buried in lipid bilayer in random distribution without any preferred orientation which results in increased motional freedom of the alkyl hydrocarbon chains in liquid crystalline state and of the polar lipid head groups,^3,57^ thus fluidizing the entire lipid bilayer which could explain why the size is increased.^56^ The particle size of liposome samples measured with DLS, in the present study, was slightly larger when compared to TEM results and this could be explained by the basis beyond these two techniques in measuring particle size. DLS puts a higher emphasis on the larger particle sizes as an intensity-based technique. However, TEM as a number-based technique shows the strong emphasis of the smallest components in the size distribution. Moreover, DLS technique measures the size of liposomes while they are in solution, whereas the TEM measures the size of dried liposomes, which is naturally smaller than that of the hydrated ones.

The magnitude of the zeta potential provides indication of the colloidal system's possible stability. As the zeta potential increases, there will be greater repulsion between particles resulting in a more stable dispersion of the colloids. If all suspended particles have high negative or positive zeta potential, then they will tend to repel each other and there will be no tendency for the particles to come together.^58^ In the present study blank NL liposomes showed negative zeta potential as (−22.38 ± 0.75 mV).

From Table 3, it should be mentioned that CL, CL-Lut and CL-Bc showed more positive zeta potential values of 36.33 ± 1.70 mV, 38.03 ± 4.78 mV and 34.40 ± 3.88 mV, respectively when compared to blank NL liposomes. The impartment of SA into liposomal membranes appears to increase the density of positive charge and hence made the zeta potential positive. These results could be attributed to the presence of SA which carries positive charge on the surface of liposomes. Generally, particles with zeta potentials more positive than +30 mV or more negative than −30 mV are normally considered stable.

Phospholipids are a group of the most studied lipids by DSC. One of their major advantages is that pure synthetic phospholipids undergo transitions at well-defined temperatures based on their structure. The pre-transition temperature (*T*_p_) is the temperature at which a transition from the gel phase to the rippled phase taking place and it is primarily related to the polar region of phospholipids. The melting of bilayer from the rippled phase to the liquid phase found at the main transition temperature (*T*_m_). The melting point (*T*_m_) is the maximum temperature of the endotherm for the lipid gel-to-fluid phase transition which has been recorded during the heating scan.^21,22^

SA addition causes a decrease in the pre-transition temperature of NL from *T*_p_ = 105 °C to *T*_p_ = 104 °C with respect to CL, indicating that the addition of positive charge caused membrane fluidization in temperature range transformation from tilted gel phase to ripple gel phase.^21^ Also, DSC demonstrated that CL had a higher main transition temperature (*T*_m_ = 151.4 °C) than that of NL (*T*_m_ = 150.4 °C), which indicated that SA had increased the membrane fluidity and affected the order of the acyl chains.^21,22^

The shift observed in the second peak related to CL after the encapsulation of lutein into the phospholipid membrane to lower temperature (from *T*_p_ = 104 °C to *T*_p_ = 102 °C), in the present study, indicating that CL-Lut had a membrane rigidisation. Additionally, the disappearance of the pre-transition is a sensitive criterion for the incorporation of substances into lipid bilayers. This may be suggested to be due to the rotation of the phospholipid polar head and the conformational changes in the lipid membrane.^57^ The shift of (*T*_p_) could be an indication of the insertion of the encapsulated lutein into the lipophilic part of the membrane's bilayer and to their contact with the polar head.

The observed decrease in the (*T*_m_) of CL-Lut could be attributed to the changes that taking place during the packing or the arrangement of the lipid membrane, in addition to the modulations that occurred in the conformation of the lipid.^16,59^ The lowered temperature of the main CL transition process indicated that the incorporation of lutein is more favorable to the formation of acyl chains in an ordered state. This suggested that lutein had a significant effect on the acyl chains of phospholipid bilayers and that its presence decreased the cooperative transition of the lipid acyl chains. Lipophilic molecules which are residing between the lipid bilayer are proposed to be able to make changes in the physical characteristics of the membranes.^59^ The incorporation of carotenoid led to decrease in the pre-transition temperature and cooperativity. The shift of the transition temperature toward lower values is larger for the pre-transition than for the main phase transition. The decreased cooperativity of the main phase transition, in the presence of carotenoid pigments, displayed by the broadening of the transition-related maxima in the thermograms, suggest that carotenoid–lipid interaction resulted in more rigidity of the fluid phase and more fluidity of the ordered phase.^15^

Lutein, having two hydroxyl groups in its ring is oriented rather perpendicularly to the plane of the membrane as these polar groups can create hydrogen bonds with the lipid headgroups. On the other hand, beta-carotene having no polar atoms in its structure, cannot interact with liposomes head groups and therefore its orientation in the membrane is less defined, and it creates higher disorder in the well-ordered fatty acids chains in the gel state of the membrane. Lutein able to fit into the phospholipid lamellar structure and must be parallelly aligned to the hydrocarbon chains in the phospholipid bilayer. Its two polar ends are contacting the opposite polar zone of the bilayer, this may lead to a very high miscibility with the phospholipid bilayer. The incorporation of beta-carotene (non-polar) into the membrane may increase the freedom of the polar head groups to move by the generation of free space in that portion of the bilayer. The main effect of beta-carotene is making the membrane less compact in its polar region.^55,60^ The incorporation of beta-carotene into liposomes did not induce a significant shift to the characteristic endothermic peak of pure liposomes. The change in phase transition indicates that the incorporated carotenoid can be localized near the interface region and within the hydrophobic core led to the carotenoid enriched microdomains.

**Fig. 8:**
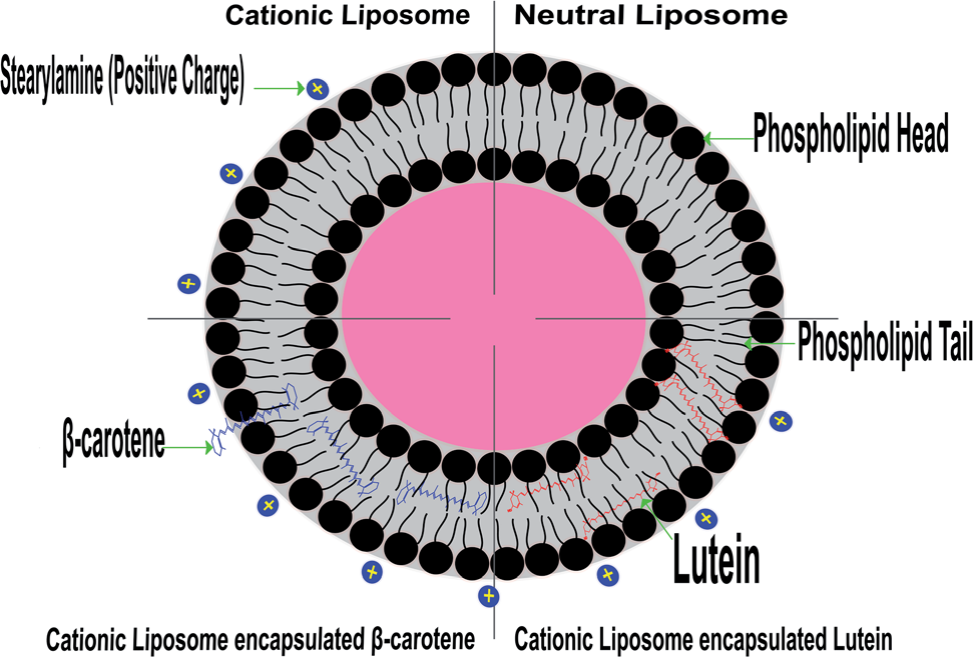
Model representation of lipid membrane organization containing lutein and beta-carotene.

The random distribution of the nonpolar beta-carotene in the lipid bilayer, without any preferred orientation (see the model presented in Fig. 8), results in increased motional freedom of the alkyl hydrocarbon chains in the liquid crystalline state and of the polar lipid head groups, thus fluidizing the entire lipid bilayer.^56^ Contrarily, the polar carotenoids, xanthophylls, like lutein orientated perpendicular to the bilayer plane, protruding through the whole lipid bilayer and anchoring the hydroxyl groups at the opposite head group polar regions, leads to a considerable reduction of membrane fluidity and has a general rigidifying effect.^15^ These different effects of carotenoids lead to the suggestion that these pigments could control the membrane fluidity of prokaryotic organisms.

The modifications that were observed in the present DSC results are confirmed by the FTIR findings, which used to detect any changes in the liposomal membrane structure by analyzing the frequency of different vibrational modes. FTIR spectroscopy was used to analyze possible changes in the structure of liposomes by analyzing the frequency of different functional groups investigating the acyl chains and head group region of the lipid molecule in presence or absence of the carotenoids.

Imparting of positive charge to liposomes formulation affects only two characteristic peaks, the symmetric CH_2_ stretching, and ^+^N–CH_3_ symmetric deformation bands. The symmetric CH_2_ stretching bands in the acyl chain at 2837.739 cm^−1^ of NL changed to 2847.3814 cm^−1^ in case of CL which indicating that SA addition creates a conformational disorder change within the acyl chains of phospholipids in the gel phase.^33–35^ Even though ^+^N–CH_3_ symmetric deformation band shows a shift towards higher wavenumber from 1400.067 cm^−1^ of NL to 1401.0312 cm^−1^ in case of CL and was not changed after the incorporation of lutein or beta-carotene into liposomes, possibly due to the electrostatic repulsive force between the positive charge of SA with choline positive charge.^35,38^

Encapsulation of lutein or beta-carotene into the cationic liposomes persuaded the change in the frequency of the symmetric CH_2_ stretching bands in the acyl chain appeared from 2847.3814 cm^−1^ of CL to 2844.49 and 2838.7 cm^−1^ for CL-Lut and CL-Bc, respectively, suggesting that lutein or beta-carotene create a conformational order change within the acyl chains of phospholipids in the gel phase.^33–35^ The wavenumber value of C=O group is shifted to lower values (from 1735.62 cm^−1^ to 1729.83 and 1721.16 cm^−1^) for CL-Lut and CL-Bc, respectively, with evidence of hydrogen bonding formation. The degree of hydrogen-bond formation was monitored in the glycerol backbone region of the lipid molecule by changes in the contours of the ester C=O stretching. The absorption bands of ester C=O are sensitive to perturbation in the polarity of their local environments and are affected by hydrogen bonding and other interactions. Therefore, changes in the contours of the ester C=O absorption band can often be interpreted in terms of structural and/or hydration changes of the bilayer polar/apolar interface. Therefore, any change in the spectra in this region can be attributed to an interaction between lutein or beta-carotene and the polar/apolar interfacial region of the membrane.^35,44,46^

The wavenumbers of symmetric and antisymmetric stretching vibration PO_2_^−^ at 1297.86 and 1137.3 cm^−1^ were not changed for all samples. Symmetric stretching band of choline CN–(CH_3_)_3_ at 909.27 cm^−1^ shifted to lower wavenumber 908.30 cm^−1^ for CL-Bc sample. This may be attributed to presence of new intermolecular hydrogen bond formation between beta-carotene and choline CN–(CH_3_)_3_ at the boundary surface within the liposomal assembly.^48^

Integration of beta-carotene into liposomes increases of motional freedom of the lipid polar groups in the lipid–water interface as well as of the fatty chains in the liquid crystalline state.^59^ Xanthophylls or polar carotenoids are oriented almost perpendicular to the plane of the membrane and traverse the double lipidic layer anchoring their hydroxyl groups in the polar region of the lipid headgroups, thus remaining immobilized inside the membrane.

Based on the above findings, the configuration of Fig. 8 was predicted based on the expectation that the studied carotenoids are localized in a hydrophobic environment of a membrane has experimental support from the measurements of highly detailed analytical and spectroscopic studies of these carotenoids.

## 5. Conclusion

The present study focused on the use of positively charged liposomes, due to the encouraging results obtained *in vitro* and *in vivo* experimentations. Our findings revealed specific abilities of lutein and beta-carotene to incorporate and modulate specifically the properties of cationic liposomal membrane derived from natural sources including sheep brain. The *in vitro* release results and the encapsulation efficiency revealed that lutein with the highest encapsulation efficiency (low leakage ability) showed the lowest drug release percentage which implies higher stability than beta-carotene and consequently lower leakage rate. The kinetic analysis of results revealed that lutein was adopted first-order kinetics when released from cationic liposomal forms. These results could be explained by the fact that the presence of lutein in the bilayers could modulate the membrane fluidity by restricting the movement of the relatively mobile hydrocarbon chains, reducing bilayer permeability, and decreases the efflux of the encapsulated drug, resulting in prolonged drug retention. Such properties of lutein may increase its uptake into the brain which allows its blood–brain barrier penetration and/or the delivery of such a carotenoid molecule specifically to the disease site.

Promising results (unpublished) have been obtained by using the present cationic liposome encapsulated carotenoids preparations in alleviating the pain and depression in an animal model of fibromyalgia. These results provide an evidence for the success of these preparation to reach its target inside the brain as was planned by the present study.

It was demonstrated in this work that the interaction between carotenoids and lipids in the membrane strongly depend on the structure of the carotenoid that may regulate the membrane's morphological and dynamic properties. Carotenoids as important lipophilic components of many natural membranes may modulate their physical properties and molecular dynamics, and this can be an important factor essential for the anti-disease activity of these compounds. Therefore, studies on the interactions between carotenoids and membrane constituents at the molecular level are very important in understanding the mechanism of their action.

## Conflicts of interest

The authors declare that there are no conflicts of interest. The author alone is responsible for the content and writing of this paper.

## Supplementary Material
